# Iturin A Extracted From *Bacillus subtilis* WL-2 Affects *Phytophthora infestans* via Cell Structure Disruption, Oxidative Stress, and Energy Supply Dysfunction

**DOI:** 10.3389/fmicb.2020.536083

**Published:** 2020-09-09

**Authors:** Youyou Wang, Congying Zhang, Jiao Liang, Lufang Wu, Wenbin Gao, Jizhi Jiang

**Affiliations:** College of Life Science, Institute of Life Science and Green Development, Hebei University, Baoding, China

**Keywords:** lipopeptides, Iturin A, *Bacillus subtilis*, *Phytophthora infestans*, inhibition

## Abstract

Potato late blight, caused by *Phytophthora infestans* (Mont.) de Bary, represents a great food security threat worldwide and is difficult to control. Recently, *Bacillus* spp. have been considered biocontrol agents to control many plant diseases. Here, *Bacillus subtilis* WL-2 was selected as a potent strain against *P. infestans* mycelium growth, and its functional metabolite was identified as Iturin A via electrospray ionization mass spectrometry (ESI-MS). Analyses using scanning electron microscopy (SEM) and transmission electron microscopy (TEM) revealed that Iturin A caused cell membrane disruption and an irregular internal cell structure. In addition, Iturin A triggered oxidative stress reactions similarly to reactive oxygen species (ROS) in *P. infestans* cells and caused mitochondrial damage, including mitochondrial membrane potential (MMP), mitochondrial respiratory chain complex activity (MRCCA), and ATP production decline. These results highlight that the cell structure disruption, oxidative stress, and energy supply dysfunction induced by Iturin A play an important role in inhibiting *P. infestans*. Additionally, *B. subtilis* WL-2 and Iturin A have great potential for inhibiting *P. infestans* mycelium growth and controlling potato late blight in the future.

## Introduction

Late blight, caused by *Phytophthora infestans* (Mont.) de Bary, can directly reduce or even eliminate potato production, and its outbreak results in grievous economic loss in the agriculture industry (Schepers et al., 2018). At present, controlling late blight is achieved mainly using disease-resistant varieties and spraying chemical pesticides (Van de Mortel et al., 2009). However, due to the rapid increase in the number of physiological races, a superphysiological race that can overcome a set of resistance genes (*R1*-*R11*) has emerged (Fukue et al., 2018). Additionally, as a result of the excessive use of chemicals, the resistance of *P. infestans* to chemical pesticides is becoming increasingly stronger, and late blight is becoming increasingly difficult to control. On the other hand, the overuse of pesticides has resulted in a great threat to food safety and the ecological environment (Meena and Kanwar, 2015). Surprisingly, biocontrol agents (BCAs), including microorganisms and their secondary metabolites, show promise as efficient and environmentally friendly alternatives to chemicals (Jiang et al., 2013; Meena and Kanwar, 2015).

With a broad range of antibiotic activity and low toxicity, cyclic lipopeptides (CLPs) synthesized by *Bacillus* spp. (iturins, surfactin, and fengycins, etc.) and *Pseudomonas* spp. (massetolide A and putisolvin, etc.) have been a research focus in the control of plant diseases in recent years (Li et al., 2014). CLPs consist of a peptide cycle composed of different amino acid arrangements and a lipid component composed of fatty acid chains of different lengths, and their molecular weight is approximately 1.1 to 1.5 kDa (Ongena et al., 2007). Due to their variety and number of amino acids and diversity in fatty acid chain length, CLPs have multiple varieties, such as the iturins, surfactin, massetolide A, putisolvin, and the fengycins (Kruijt et al., 2009; Van de Mortel et al., 2009; Meena and Kanwar, 2015; Luna-Bulbarela et al., 2018). Additionally, CLPs have great potential to inhibit a series of pathogenic bacteria, fungi, viruses, etc. (Meena and Kanwar, 2015). Specifically, surfactin family has powerful antibacterial activities but low antifungal activities, while the iturins family shows strong antifungal activities against *Candida albicans* (Tabbene et al., 2015), *Sclerotinia sclerotiorum* (Kumar et al., 2012), *Botrytis cinerea* (Arrebola et al., 2010), and *Fusarium graminearum* (Gu et al., 2017). Also, the main mechanisms of action of the iturins family against fungi are the increase of cell membrane permeability and reactive oxygen species (ROS) accumulation, decrease of mitochondrial membrane potential, and condensation of the cell nucleus (Tabbene et al., 2015; Lei et al., 2019). However, there are only a few reports of using CLPs to inhibit oomycetes (Ongena and Jacques, 2008; Meena and Kanwar, 2015). Among the CLPs, only the putisolvins and massetolide A families, have been verified to inhibit oomycetes *Phytophthora capsici* and *P. infestans* by zoospores inactivation and mycelia morphological distortion (Kruijt et al., 2009; Van de Mortel et al., 2009). Also, the inhibition effects as well as the mechanisms of the CLP Iturin A family on oomycete *P. infestans* are still unclear.

In our previous studies, *P. infestans* mycelium distortion and sporangium germination rate reduction were often caused by antagonistic *Bacillus* spp. and their metabolites. Unfortunately, the specific damaging mechanisms underlying these phenomena caused by antagonistic microorganisms or their CLPs metabolites in *P. infestans* are still unknown. In this study, the antagonistic effects of three bacteria including *Bacillus subtilis* WL-2 (CGMCC 17771), *Bacillus pumilus* W-7 (CGMCC 17770), and *Pseudomonas fluorescens* WL-1 (CGMCC 17769) against *P. infestans* mycelium growth were preliminarily compared. Then, *B. subtilis* WL-2 strain was selected as an efficient BCA for *P. infestans* inhibition. Additionally, CLPs Iturin A and surfactin families from WL-2 were extracted and further identified using tandem mass spectrometry (MS/MS). Most importantly, the inhibitory effects and underlying specific mechanisms of Iturin A on *P. infestans* mycelium growth, including cell structure disruption, oxidative stress, and energy supply dysfunction, were investigated. We hope that these results will increase our comprehension of the important role of Iturin A as an antioomycete agent against potato late blight.

## Materials and Methods

### Inhibition of *P. infestans* by Three Strains

The oomycete *P. infestans* (Mont.) de Bary W101 was obtained from the China General Microbiological Culture Collection Center (CGMCC 3.19919) and grown on rye (R) solid medium at 20°C in the dark. *B. subtilis* WL-2 (CGMCC 17771), *Pseudomonas fluorescens* WL-1 (CGMCC 17769), and *Bacillus pumilus* W-7 (CGMCC 17770) were isolated from *Capsicum frutescens* leaves and cultured on Luria Bertani (LB) solid medium. Living cells (LCs) of bacteria were grown on solid medium and incubated for 24 h at 37°C. To obtain a cell suspension (CS, 1 × 10^7^ CFU/mL), LB liquid medium was incubated for 20 h at 37°C (200 rpm), and the final concentration (1 × 10^7^ CFU/mL) was adjusted with distilled water. The CS was filtered through cellulose acetate membranes (0.22 μm) to obtain a cell-free supernatant (CFS). *P. infestans* mycelium was oscillated to obtain sporangium (1 × 10^7^ CFU/mL), and the sporangium was released at 10°C for 3 h to prepare a zoospore suspension (1 × 10^7^ CFU/mL). Inhibitory effects on the growth of *P. infestans* mycelium were assessed on LCs, the CS, and the CFS using the dual-culture plate method (Li et al., 2014). First, a mycelium disk (diameter = 7 mm) was placed on the center of R solid medium (diameter = 9 cm) and cultivated for three days. Then, LCs were placed 3 cm away from the disk, and blank LB medium was placed as a control. Similarly, according to the punch method (Ding et al., 2017), every punch (9 mm) was added to 100 μL of CS or CFS, and an equal volume of blank LB liquid medium was added as a control. Different from the LCs group, in the CS treatment, the bacteria cells remained viable in LB liquid medium (the suitable medium for bacterial culture) with a concentration above 1 × 10^5^ CFU/mL even after 5 days confrontation. As for the treatment of CFS, there was no bacteria cells and without pathogen (*P. infestans*) induction. Finally, after coincubation at 20°C for five days, the inhibitory zones and the inhibition rates were determined by the following formula:

Inhibition rate (%) = (C−T)/C × 100 (Ding et al., 2017).

where C represents the colony radius of the control, and T is the radius of the treatment group.

### MALDI-TOF-MS Analysis and Antagonism Assay of Crude Lipopeptide Extract (CLE)

CS (2 mL) was transferred into a flask containing 400 mL of Landy liquid medium and cultured at 30°C and 180 rpm for 96 h to accumulate CLPs (Arrebola et al., 2010). According to the acid precipitation method (Zhang and Sun, 2018), the CLPs were precipitated and dissolved in methanol, and a rotary evaporator (RE52CS-1, Yarong, Shanghai, China) was used to obtain CLE for further analysis. Matrix-assisted laser desorption ionization time-of-flight mass spectrometry (MALDI-TOF-MS, AutoFlex III, Bruker Daltonics, United States) was utilized to analyze the classification of CLE (1 mg/L, 10 μL) in positive mode and at 20 kV accelerating voltage, and compounds with molecular weights from 800 to 1,700 were analyzed (Yang et al., 2015; Fan et al., 2017).

The disk diffusion method (Medeot et al., 2017) was adopted to evaluate the CLE antioomycete activity. *P. infestans* disks (7 mm) were incubated on R solid medium plates for three days, and paper disks (5 mm) containing 6 μL of CLE solution (1, 3, and 5 mg/mL) were then placed. Meanwhile, the same volume of the fungicide metalaxyl (15 μg/mL) and methanol solution were used as controls. After coincubation at 20°C for five days, the inhibition rates were determined (Ding et al., 2017).

### HPLC Purification of Lipopeptides and MALDI-TOF-MS/MS Analysis

Standard lipopeptides (surfactin and iturins, Sigma-Aldrich, United States) and CLE solution (10 mg/L) were run separately on an HPLC system (Waters, E2695, United States) with a C_18_ reverse-phase column (5 μm, 4.6 × 150 mm) under the same conditions (Yang et al., 2015). A solution of water and acetonitrile in a ratio of 20:80 by volume was selected for the mobile phase. The injection rate was 1 mL/min, and the eluate was monitored at 214 nm. According to the retention time of standard lipopeptides, the peaks of potential CLPs contained in the CLE were collected (Yang et al., 2015; Fan et al., 2017).

As described in the MALDI-TOF-MS method, 1 μg/mL purified lipopeptides (peaks a and b from HPLC) were examined using MALDI-TOF-MS/MS (MALDI-TOF, AUTOFLEX III, Bruker Daltonics, United States) in collision-induced dissociation (CID) mode to clarify the amino acid sequences of the CLPs (Yang et al., 2015). Depending on the precursor ions of interest, a suitable collision energy between 35 and 50 eV was selected (Gong et al., 2015).

### Antagonism Analysis of Purified Surfactin and Iturin A

The antioomycete activity of purified surfactin and Iturin A was evaluated using the disk diffusion method (Medeot et al., 2017), and 6 μL of CLPs at different concentrations (20, 30, 40, and 50 μg/mL) were added to the paper disks (5 mm). Additionally, distilled water was used as a control. Finally, after coincubation at 20°C for five days, the inhibitory rates were determined the same above. Marginal *P. infestans* mycelium disks inhibited by Iturin A (different concentrations) at 20°C for five days were transferred onto fresh R solid medium (without Iturin A solutions) to recover growth, and an uninhibited mycelium disk was treated as a control (Wang et al., 2020). All the plates were incubated at 20°C for seven days, and the mycelium relative recovery growth rates were calculated according to the formula below:

Relative recovery growth rate (%) = (colony diameter in treatment group/colony diameter in control) × 100.

In addition, *P. infestans* mycelium inhibited by Iturin A (different concentrations) at 20°C for twelve days was collected with ice-cold sterile water and oscillated to obtain sporangia solution (1 × 10^7^ CFU/mL) (Bruisson et al., 2019; Wang et al., 2020). Then, the inhibited sporangia were recovered to release zoospores at 4°C for 3 h and maintained at 25°C in the dark for 5 h to complete sporangium direct germination (Hunziker et al., 2015; Bruisson et al., 2019). Also, the sporangia solution (1 × 10^7^ CFU/mL) from the mycelium incubated without Iturin A was treated similarly as the control. Finally, an optical microscopy (OM) system (BX53, OLYMPUS, Japan) was used to observe 300 sporangia to calculate the release and direct germination rates via the formula below:

Release or germination rate (%) = (total release or germination number/number of total spores) × 100.

### Inhibition of *P. infestans* by Iturin A

#### Scanning Electron Microscopy (SEM) and Transmission Electron Microscopy (TEM)

Marginal mycelium inhibited by Iturin A (50 μg/mL) was collected and fixed using 2.5% (v/v) glutaraldehyde (Solarbio, Beijing, China) for 24 h and dehydrated for 30 min after fixing using aqueous ethanol solutions (30%, 50%, 70%, and 90%, v/v). Then, mycelium morphological and surface changes were observed using an SEM system (JSM-7500F, JEOL, Japan) (Cui et al., 2016). A TEM system (JEM-2100F, JEOL, Japan) was also adopted to evaluate the structural characteristics of inhibited mycelium. Similar to the method above, 2.5% (v/v) glutaraldehyde was used to fix mycelium, and 1% (v/v) osmium tetroxide was also used to fix mycelium at 20°C for 20 min. Finally, a microtome (YD335, Leica, Germany) was used to prepare thick specimens (70 nm) for TEM observation (Cui et al., 2016; Huiskonen, 2018).

### *P. infestans* Cell Membrane Damage

*P. infestans* mycelium and sporangia inhibited by Iturin A (50 μg/mL) were collected, and then 30 μM propidium iodide was used to stain cells in an ice bath for 10 min. An uninhibited group was used as a control, and the mycelium was observed using a filter at 535 nm/615 nm in a confocal fluorescence microscopy (CFM) system (FV3000, Olympus, Japan) (Zhao et al., 2014). Changes in membrane permeability caused by Iturin A were investigated using mycelium-rich solution based on the changes in the electrical conductivity and changes in the optical density at 260 nm and 280 nm. First, *P. infestans* mycelium (100 mg) inhibited by Iturin A (50 μg/mL) was suspended in distilled water (20 mL). Additionally, uninhibited mycelium was treated as a control. The cell membrane permeability was determined using a conductivity meter (S7-Meter, Mettler Toledo, Switzerland) based on the electrical conductivity of the mycelium solution after suspension for 0, 20, 40, 60, 80, and 100 min, respectively. The conductivity of a mycelium solution boiled for 10 min was considered the final conductivity. Finally, the relative conductivity was calculated according to the following formula:

Relative conductivity (%) = (conductivity/final conductivity) × 100 (Li et al., 2016).

The absorbances of mycelium solutions at 260 and 280 nm were measured by an ultraviolet-visible light detector (UV-1800, Shimadzu, Japan) to assess nucleic acid and protein leakage (Dolezalova and Lukes, 2015).

### Accumulation of Reactive Oxygen Species (ROS) and Malondialdehyde (MDA) Production

Three *P. infestans* mycelium disks were transferred into R liquid medium (100 mL) and cultured at 20°C and 180 rpm for 48 h. Afterward, Iturin A (50 μg/mL, final concentration) was added and incubated for 0, 4, 8, 12, 16, 20, and 24 h, and the ROS-inducing drug Rosup (Solarbio, Beijing, China) at 10 μg/mL (final concentration) was incubated with the mycelium culture for 20 min as a positive control. Subsequently, *P. infestans* mycelium (100 mg) from different groups was coincubated with 2′,7′-dichlorodihydrofluorescein diacetate (DCFH-DA) (10 μM) for 20 min, and the CFM system was used to analyze the mean fluorescence intensity (Tian et al., 2018). Additionally, the MDA concentration, a marker of lipid peroxidation, was analyzed in *P. infestans* mycelium (100 mg) from different groups using the MDA assay kits (Beyotime, Shanghai, China) and an ultraviolet-visible light detector measuring the absorbance at 532 nm (Dolezalova and Lukes, 2015).

### Mitochondrial Damage and Energy Supply Dysfunction

JC-1 was used to assay the mitochondrial membrane potential (MMP, mtΔψ) in *P. infestans* mycelium (Pushpanathan et al., 2013). Based on the results above, the ROS generation induced by Iturin A was highest when the incubation time was 16 h; therefore, the mycelium incubated for 16 h was collected and stained with 10 μg/mL JC-1 (Beyotime, Shanghai, China) in the dark for 20 min. Next, red and green fluorescence was monitored at Ex/Em = 490/525 nm and 490/590 nm using a CFM system (Pushpanathan et al., 2013). The mitochondrial respiratory efficiency was determined from the mitochondrial respiratory chain complex activity (MRCCA), respiratory control rate (RCR), and oxidative phosphorylation efficiency (P/O). After inhibition, 5 mL of lysis buffer was mixed with mycelium to extract mitochondria according to the Mitochondrial isolation kit (Beyotime, Shanghai, China) instructions (Sun et al., 2019). The MRCCA, including the activities of complexes I-V, was measured based on the absorbance decline at different wavelengths (Davies et al., 2001; Schägger and Pfeiffer, 2001). The rate of NADH oxidation catalyzed by complex I was evaluated at 340 nm to reflect complex I activity. In addition, 2,6-dichlorophenol indophenol (DCPIP) was used as a coloring agent, and the absorbance reduction at 600 nm was considered the decline in the activity of complex II. Complex III activity was detected according to the rate of ferricytochrome c reduction by CoQ_2_ (absorbance at 550 nm), and complex IV activity was evaluated as the cyanide-sensitive oxidation of ferrocytochrome c (absorbance at 550 nm). Additionally, complex V activity was determined by measuring the rate of NADH oxidation (absorbance at 340 nm). Uninhibited mycelium was treated as a control.

The inhibited mycelium was placed in a respirator (O2k-FluoRespirometer, Oroboros, Austria) pool containing 2 mL of respiratory solution. Then, 2 mol/L glutamic acid (10 μL), 0.4 mol/L malic acid (5 μL), and 2.5 mmol/L succinic acid (100 μL) were added into the reaction pool; subsequently, 2 μL of 100 mmol/L adenosine diphosphate was added to obtain state 3 respiration. At the time of ADP depletion, the respiratory rate was considered state 4. The ratio of state 3 to state 4 was considered the RCR (Amaroli et al., 2019; Sun et al., 2019). The ratio of ATP production to oxygen production in the presence of respiration substrates and ADP was considered the P/O (Hinkle, 2004). Uninhibited mycelium was assayed as a control.

### Statistical Analysis

All the treatments were repeated three times, and the final results were shown as the average values. Statistical analysis, especially to identify significant differences, was carried out with SPSS software (version 22, IBM, United States). The data were subjected to one-way analysis of variance (ANOVA), and significant differences were detected using the least significant difference (LSD) of means test at *P* < 0.05.

## Results

### Comparison of the Inhibition of *P. infestans* by the Three Strains

The LCs of the three strains had a strong inhibitory effect on the growth of *P. infestans* mycelium, and all inhibition rates were above 60% (Supplementary Figure S1 and Table 1). The WL-2 strain had the strongest inhibitory effect, and its inhibition rate reached a maximum of 75.6%, which was significantly different from that of the other strains (*P* < 0.05, Table 1). The suppression rates of CS treatment were all above 80%, the inhibitory effect of WL-2 was the most prominent, and its inhibition rate reached a maximum of 93.7% (Supplementary Figures S1A,B and Table 1). Additionally, in the CFS experiment, the inhibition effect of the WL-2 strain (inhibition rate of 80.7%) was significantly better than that of WL-1 and W-7 (*P* < 0.05, Table 1).

**TABLE 1:** Comparison of the inhibition of *P. infestans* by the three strains.

Strains	Inhibition rates (%)		
	
	LCs	CS	CFS
WL-2	75.6 ± 2.85 a	93.7 ± 2.91 a	80.7 ± 2.49 a
WL-1	65.4 ± 1.72 b	86.1 ± 2.92 b	56.6 ± 1.70 b
W-7	62.5 ± 2.01 b	84.2 ± 2.51 b	58.7 ± 1.79 b
CK	0.0 ± 0.00 c	0.0 ± 0.00 c	0.0 ± 0.00 c

*LCs: Living cells, CS: Cell suspension, CFS: Cell-free supernatant. The different letters a, b, c, and d in the same column symbolize a significant difference (*P* < 0.05). Data are expressed as the average of three replicates ± standard deviation.*

### MALDI-TOF-MS and Antagonism Assays of CLE

**FIGURE 1:**
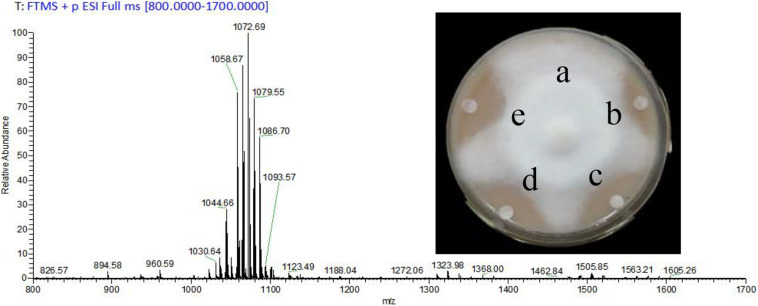
Analysis of crude lipopeptide extract (CLE) from *B. subtilis* WL-2 using MALDI-TOF-MS and its inhibitory effect on *P. infestans* mycelial growth. a: Control (distilled water), b–d: CLE at 1, 3, and 5 mg/mL, respectively, e: Metalaxyl (15 μg/mL).

The yield of prepared CLE was 2.3 g/L, and lipopeptides surfactin and Iturin A were preliminarily clarified in the CLE. The most abundant molecular weights of 1,044.66, 1,058.67, 1,072.69, and 1,086.70 were speculated to be surfactin (C_14_ - C_17_) with Na^+^ adduct ions (Figure 1). The ion peak at *m/z* 1,079.55 was considered Iturin A (C_15_) with Na^+^ adduct ions (Figure 1). As the CLE concentration increased from 1 to 5 mg/mL, the inhibition rates also expanded from 38.6% (Figure 1b) to the maximum of 80.2% (Figure 1d).

### CLE Purification Using HPLC and MS/MS Analysis

The commercial lipopeptides produced two obvious peaks at 21.6 min (peak c, surfactin) and 23.2 min (peak d, iturins, Supplementary Figure S2). The corresponding peaks of the CLE, including peak a at 21.4 min and peak b at 23.6 min, were collected (Supplementary Figure S2).

The obvious MS signals from peak a ranging from *m/z* 1,000 to 1,100 were hypothesized to be surfactin with C_14_ to C_17_ fatty acid chains (Supplementary Figure S3A and Table 2). Supplementary Figure S3C illustrated the MS/MS spectrum of surfactin C_14_, with a peak at *m*/*z* 1,044.66 [M+Na]^+^. The series of b^+^ ions at *m*/*z* 931→818→703→604→378 (-H_2_O, 360) signified the loss of Leu, Asp, Val, and Leu - Leu/Ile, respectively, at the peptide bonds, and the ion at *m*/*z* 360 corresponded to the C terminus of a β-OH fatty acid combined with Glu. Starting from the y^+^ end, the ions at *m/z* 267→481→594→707 represented the cleavage of the peptide bonds connecting Leu/Ile - Leu, Asp - Val, Leu, and Leu/Ile, respectively, so the ion at *m/z* 707 corresponded to the total mass of ion fragments containing Leu/Ile - Leu - Val - Asp - Leu - Leu/Ile. The MS/MS results confirmed that surfactin C_14_ was β-OH fatty acid - Glu - Leu/Ile - Leu - Val - Asp - Leu - Leu/Ile (Supplementary Figure S3C). The structure of surfactin C_15_, with a peak at *m*/*z* 1,058.67 [M+Na]^+^, was determined from the results in Supplementary Figure S3D. Similar to the above discussion, the series of y^+^ ions at *m*/*z* 154→267→382→481→594→707 represented the sequential connections between amino acids Leu/Ile, Leu, Asp, Val, Leu, and Leu/Ile, respectively. For the b^+^ fragments, the ions at *m/z* 945→832→717→618→391 illustrated the loss of Leu, Asp, Val, and Leu - Leu/Ile, respectively, and the ion at *m*/*z* 391 corresponded to a β-OH fatty acid connected to Glu (Supplementary Figure S3D). Surfactin C_16_, at *m*/*z* 1,072.69 [M+Na]^+^, exhibited y^+^ fragment ions that were the same as above, with the sequence of Leu/Ile - Leu - Val - Asp - Leu - Leu/Ile at the N terminus (Supplementary Figure S3E). In terms of the b^+^ results, the most significant ion, at *m*/*z* 406 (-H_2_O, 388), confirmed that a β-OH fatty acid (C_16_) was connected to Glu (Supplementary Figure S3E). Additionally, the y^+^ fragment ions found at *m*/*z* 1,086.69 (C_17_, Supplementary Figure S3F) signified that the same peptide connection was present in surfactin C_14–16_. The b^+^ fragment ions at *m/z* 973 and 860 corresponded to the sequence β-OH fatty acid (C_17_) - Glu - Leu/Ile - Leu - Val - Asp - Leu. In summary, the MS/MS peaks at *m*/*z* 1,044.66, 1,058.67, 1,072.69, and 1,086.69 were the same for the entire surfactin subfamily but differed by 14 Da (-CH_2_-).

**TABLE 2:** MS/MS detection of potential lipopeptides collected from peaks after HPLC purification.

Lipopeptide	Fatty acid chain	Molecular formula	Calculated (*m/z*)		
			
			[M+H]^+^	[M+Na]^+^	[M+K]^+^
Surfactin (peak a)	C_14_	C_52_H_91_N_7_O_13_	1,022.68	1,044.66	-
	C_15_	C_53_H_93_N_7_O_13_	1,036.69	1,058.67	-
	C_16_	C_54_H_95_N_7_O_13_	-	1,072.69	1,088.66
	C_17_	C_55_H_97_N_7_O_13_	-		1,102.68
Iturin A (peak b)	C_14_	C_48_H_74_N_12_O_14_	1,043.55	1,065.54	-
	C_15_	C_49_H_76_N_12_O_14_	1,057.57	1,079.55	-

*The retention time of peak a and b from HPLC system was at 21.4 and 23.6 min, respectively.*

Peak b consisted of intense signals at *m/z* ranging from 1,000 to 1,100 and signified ions characteristic of Iturins A C_14_ to C_15_ (Supplementary Figure S3B and Table 2). The MS/MS spectrum of Iturin A at *m*/*z* 1,065.54 [M+Na]^+^ was shown in Supplementary Figure S3G, and the b^+^ fragment ions at *m/z* 186→300→428 represented the sequence Tyr - Asn - Gln. In addition, the series of y^+^ ions at *m*/*z* 563→449→362 signified the cleavage and loss of Asn, Asp, and Ser, respectively, and the ions at *m*/*z* 563 represented the sequence Asn - Ser - β-OH fatty acid (C_14_) - Asn (Supplementary Figure S3G). Supplementary Figure S3H showed the detection of Iturin A at *m*/*z* 1,079.55 [M+Na]^+^. The y^+^ fragment ions at *m*/*z* 300→414→653 symbolized the sequential connections between Tyr - Asn, Asn, and a β-OH fatty acid (C_15_) (Supplementary Figure S3H). In addition, the b^+^ ion fragments in the order *m*/*z* 248, 362, 449 (-H_2_O, 431), and 670 illustrated the sequence Gln - Pro - Asn - Ser - β-OH fatty acid (C_15_) (Supplementary Figure S3H). The results above demonstrated the presence of Iturin A (C_14_ and C_15_) with the structure β-OH fatty acid - Asn - Tyr - Asn - Gln - Pro - Asn - Ser.

### Antiactive Effects of Purified Surfactin and Iturin A on *P. infestans* Mycelium

**FIGURE 2:**
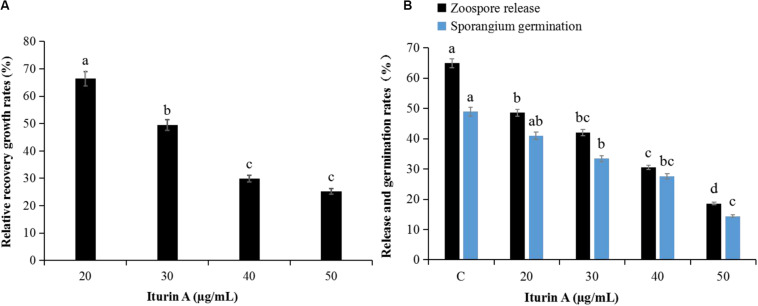
Recovery of *P. infestans* mycelium and sporangia after inhibition by Iturin A (concentrations at 20, 30, 40, and 50 μg/mL, respectively). **(A)** Relative recovery growth rates of mycelium, **(B)** Zoospore release and direct germination rates of sporangium after inhibition. The capital letter C means the control group, and the different lowercase letters indicate a significant difference between the different groups at the level of *P* < 0.05.

The antioomycete results showed that surfactin did not inhibit mycelium growth, and there were no obvious inhibition zones even at 50 μg/mL (Supplementary Figure S4A). However, the inhibitory effect was clearly dependent on the concentration of Iturin A (Supplementary Figure S4B). Iturin A at 50 μg/mL produced a maximum inhibition rate to 84.9% (Supplementary Figure S4Be). The recovery of *P. infestans* mycelium (Figure 2A and Supplementary Figure S4C) and sporangium (Figure 2B) after inhibition was determined. After inhibition by Iturin A (20, 30, 40, and 50 μg/mL), mycelium relative recovery growth rates were 66.4%, 49.5%, 29.9%, and 25.2%, respectively, which signified that the concentrations of Iturin A were positively correlated with the degree of mycelium damage (Figure 2A and Supplementary Figure S4C). Moreover, as the inhibition concentrations of Iturin A increased from 20 to 50 μg/mL, the zoospore release and sporangium direct germination rates declined significantly. Additionally, the zoospore release rates declined from 64.9% to 18.6% (lowest), and the direct germination rates decreased from 48.9% to 14.4% (lowest, Figure 2B). In addition, all the lowest rates were significantly different from those of the control (64.9% and 48.9%, *P* < 0.05, Figure 2B).

### Inhibition of *P. infestans* by Iturin A

#### Observation Using SEM and TEM

**FIGURE 3:**
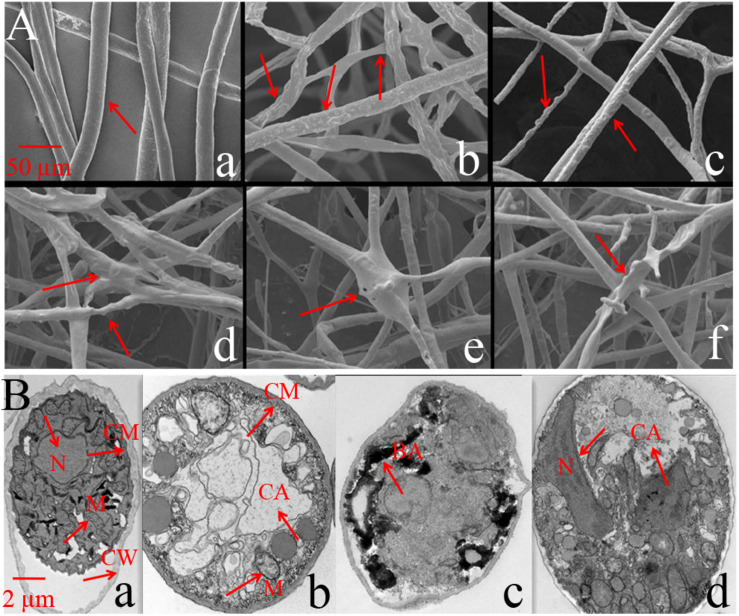
*Phytophthora infestans* mycelium deformation after Iturin A (50 μg/mL) inhibition. **(A)** SEM observation, a: Straight and smooth mycelia (control), b–f: Iturin A groups, where mycelia were rough, bulged, uneven, and abnormally expanded in branches. **(B)** TEM observation, a: Mycelium growth with intact cell membrane (CM), cell wall (CW), and normal organelle arrangement, b–d: Iturin A groups with disrupted cell membranes, disordered organelles, sparse mitochondria (M) with few ridges, a large cavitation area (CA), black aggregates (BA), and shifted nuclei (N).

SEM results showed that the mycelium in the control was straight and smooth without any expansion (Figure 3Aa). However, after treatment with Iturin A (50 μg/mL), the mycelium was rough and uneven on the surface (Figures 3Ab,c) and the mycelium was locally raised with an uneven width (Figures 3Ac,d). In addition, some mycelium twisted into clusters and formed unusual bulges (Figures 3Ad,e), and even some abnormal expanded branches appeared in parts of the mycelium (Figure 3Af). TEM was used to examine the structural changes within cells. TEM results showed that the normal mycelial cell membrane was intact, organelles were distributed in a normal arrangement, and mitochondria, including inner ridges, were abundant (Figure 3Ba). After treatment with Iturin A (50 μg/mL), the mycelial cell membrane was disrupted, mitochondria and ridges were sparse (Figure 3Bb), and even a large area of cavitation appeared in the center of the cytoplasm (Figures 3Bb,d). Additionally, irregular organelle shapes with unclear boundaries and obvious black aggregates were visible in some cells (Figure 3Bc). In the treatment group, the mycelial nuclei affected by cavitation shifted to the cell edge (Figure 3Bd).

#### Effects of Iturin A on Cell Membrane Integrity

**FIGURE 4:**
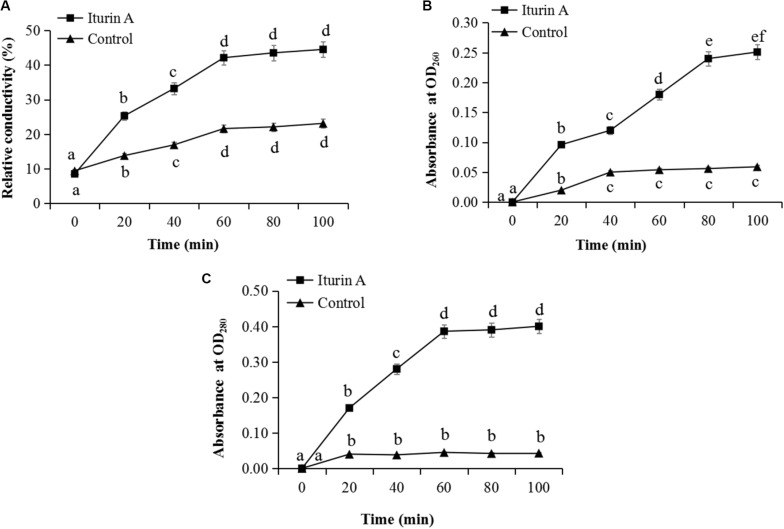
Effects of Iturin A (50 μg/mL) on *P. infestans* cell membrane permeability. **(A)** Relative conductivity, **(B)** Nucleic acid leakage, **(C)** Protein leakage. The lowercase letters a, b, c, d, e, and f indicate a significant difference at the level of *P* < 0.05.

The membrane integrity results (Supplementary Figure S5) showed that after treatment with Iturin A (50 μg/mL), hyphae (Supplementary Figure S5Ab) and sporangia (Supplementary Figure S5Ad) displayed obvious red fluorescence, indicating that Iturin A could cause substantial cell membrane defects. Moreover, the red fluorescence rates of sporangium were approximately 68% in the treatment group but only 21% in the control, which were significantly different values (*P* < 0.05). Cell membrane permeability changes reflected by the relative conductivity are shown in Figure 4A. The relative conductivity of the control had increased from 9.7% to 19.6% at 60 min; however, in the Iturin A group, the conductivity improved from 10.2% at the beginning to 41.8%. The maximum relative conductivity of the treatment group (44.6%) was twice as high as that of the control (20.9%), and these values were significantly different (*P* < 0.05, Figure 4A). Regarding the leakage of nucleic acids, the absorbance reached a maximum of 0.251 (100 min) and was significantly higher than that of the control (highest absorbance at 0.059, *P* < 0.05, Figure 4B). Regarding protein leakage, the highest absorbance (0.410) in the Iturin A group was significantly higher than that of the control (highest absorbance of 0.038, *P* < 0.05, Figure 4C).

#### ROS Reaction and MDA Production

**FIGURE 5:**
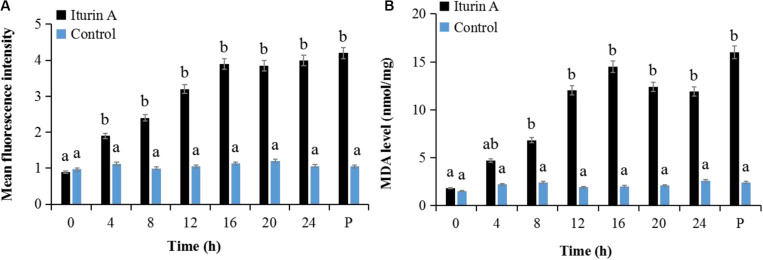
Reactive oxygen species (ROS) generation and malondialdehyde (MDA) production in *P. infestans* cell after Iturin A inhibition. **(A)** Mean fluorescence intensity of DCFH-DA (2′, 7′-dichlorodihydrofluorescein diacetate) for ROS detection, **(B)** MDA production. The capital letter P indicates the positive control (Rosup), and the lowercase letters a and b indicate a significant difference (*P* < 0.05) within the same treatment group.

Cell oxidative stress presented by ROS reaction was measured using DCFH-DA staining (Kobayashi et al., 2002). Malondialdehyde (MDA) production indicating the lipid peroxidation due to ROS accumulation and cell damage (Dolezalova and Lukes, 2015) was also evaluated as a marker of oxidative stress. As the Iturin A (50 μg/mL) treatment time increased, the mean fluorescence intensity of DCFH-DA obviously increased (Figure 5A and Supplementary Figure S6). Specifically, the fluorescence intensity in the Iturin A group was significantly higher than that in the control after 4 h (*P* < 0.05, Figure 5A). Additionally, the highest fluorescence intensity was four times higher than that in the control (16 h, *P* < 0.05), and there was no significant difference from that of the positive control (*P* > 0.05, Figure 5A and Supplementary Figure S6). In addition, the concentrations of MDA from 8 h to 24 h in the Iturin A group were significantly higher than those in the control (approximately 2 nmol/mg, *P* < 0.05) and reached a maximum of 15.2 nmol/mg after 16 h coinoculation, which was not significantly different from the maximum value of the positive control (16.1 nmol/mg, *P* > 0.05, Figure 5B).

#### Mitochondrial Damage and Energy Supply Dysfunction

**FIGURE 6:**
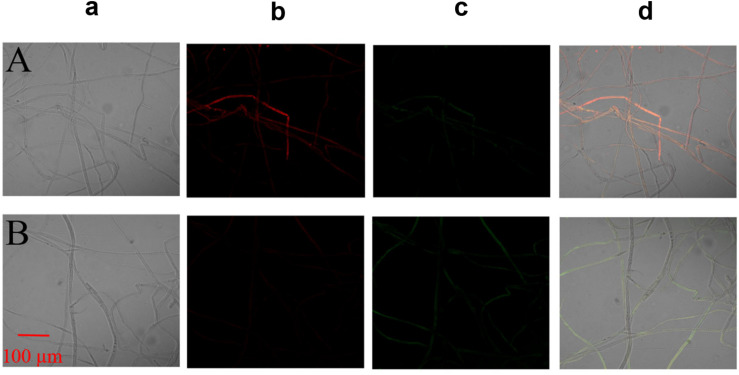
Effect of Iturin A on the mitochondrial membrane potential (MMP) of *P. infestans* mycelium. **(A)** Control, **(B)** Iturin A (50 μg/mL) inhibition for 16 h. a: Optical channel, b: Red fluorescence channel, c: Green fluorescence channel, d: Red and green channels merged.

**FIGURE 7:**
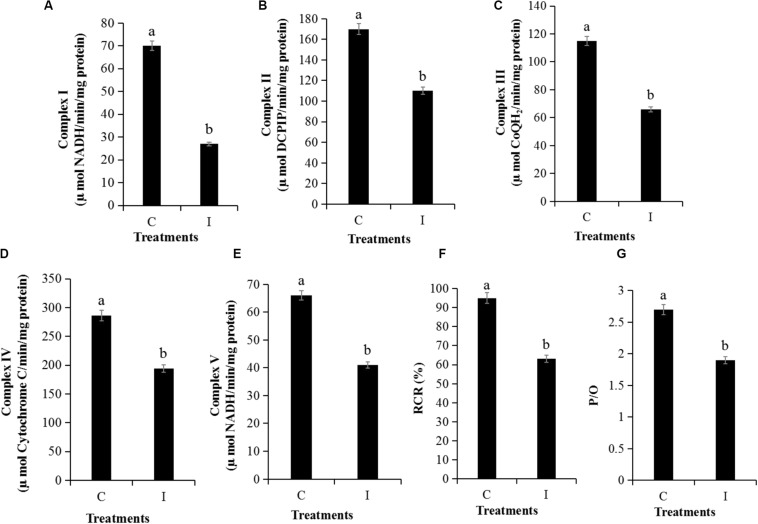
Detection of mitochondrial respiratory activity of *P. infestans* cell after Iturin A (50 μg/mL) inhibition for 16 h. **(A–E)** Mitochondrial respiratory chain complexes activity (MRCCA) of I to V, respectively, **(F)** Respiratory control rate (RCR), **(G)** Oxidative phosphorylation efficiency (P/O). The capital letters C and I represent the control and Iturin A treatment groups, respectively, and the different lowercase letters indicate a significant difference (*P* < 0.05).

The MMP assay using JC-1 fluorescence was shown in Figure 6. The control group exhibited obvious red fluorescence of J-aggregates in mitochondria (Figures 6Ab,d). Compared with the control group, the Iturin A-treated mycelium displayed dramatically changed green fluorescence (Figures 6Bc,d), which indicated that Iturin A could cause a decrease in the MMP. After treatment with Iturin A (50 μg/mL), the mitochondrial respiratory effect reflected by the indices MRCCA, RCR, and P/O was detected, and it was shown in Figure 7. MRCCAs I-V in this group were all significantly different from those of the control group (*P* < 0.05) and reduced remarkably to approximately 61%, 35%, 43%, 31%, and 38% of the control values, respectively (Figures 7A–E). The RCR and P/O values in the control group were 95% and 2.7, respectively, which were significantly higher than those in the Iturin A group (63% and 1.9, respectively, *P* < 0.05, Figures 7F,G).

## Discussion

The oomycete *P. infestans* is the culprit behind potato late blight, causing the largest economic losses for potato crops (Schepers et al., 2018). Control of late blight using BCAs, including microorganisms and secondary metabolites, could be an effective measure to address the problems of food safety, environmental protection, and disease resistance resulting from chemicals (Meena and Kanwar, 2015). Some *Bacillus* species are considered the best potential candidates because of their ability to survive in various environments and their variety of biocontrol molecules (Raaijmakers et al., 2010). In this article, we found that *B. subtilis* WL-2 had the greatest potential to inhibit *P. infestans* mycelium among the three species of bacteria. Ongena and Jacques (2008) once reported that CLPs with a wide range of antimicrobial activities were the most common metabolites of some *Bacillus* species. In this study, we used MS/MS analysis to confirm that the WL-2 strain could also produce the CLPs surfactin and Iturin A subfamilies. Additionally, the structural formula of the surfactin was β-OH fatty acid - Glu - Leu/Ile - Leu - Val - Asp - Leu - Leu/Ile with a fatty acid chain from C_14_ to C_17_ in length, and the Iturin A (C_14_ - C_15_) had a structure of β-OH fatty acid - Asn - Tyr - Asn - Gln - Pro - Asn - Ser.

The iturins family is known to exhibit direct antagonistic activities against *C. albicans* (Tabbene et al., 2015), *Aspergillus flavus* (Moyne et al., 2001), *S. sclerotiorum* (Kumar et al., 2012), *B. cinerea* (Arrebola et al., 2010), *Monilinia fructicola* (Arrebola et al., 2010), and *F. graminearum* (Gu et al., 2017); however, there has been relatively little reports focused on the inhibition of oomycetes by iturins, and the inhibition effects as well as the mechanisms of the CLP Iturin A family on oomycete *P. infestans* are still unclear (Ongena and Jacques, 2008; Meena and Kanwar, 2015). In this study, we demonstrated that after inhibition by Iturin A, the inhibition zone of *P. infestans* mycelium reached a maximum inhibition rate of 84.9%. With Iturin A inhibition, the lowest rates of *P. infestans* zoospore release and direct sporangium germination were only 18.6% and 14.4%, respectively. All these results mainly corresponded to the Van de Mortel et al. (2009) report that after exposure to CLP massetolide A (10 μg/mL), *P. infestans* mycelium and sporangia production declined by approximately 50%, and the sporangium germination activity almost disappeared. Similarly, other reports showed that the CLP fengycin family exhibited zoosporicidal activities against *Phytophthora* spp. through spore swarming mobility inhibition and membrane solubilization (De Bruijn et al., 2007; Kruijt et al., 2009).

*P. infestans* inhibited by Iturin A must be in a damaged state, but the specific molecular mechanisms of this injury remain unclear. Previous reports signified that iturins produced by *Bacillus* species exhibited obvious antifungal mechanisms involving cell membrane integrity disruption and organelle damage (Kumar et al., 2012; Gu et al., 2017). A similar report stated that *F. graminearum* mycelium affected by iturins displayed severe morphological changes, including mycelium distortions, cell membrane leakage, and plasma membrane separation from the cell wall (Gong et al., 2015). In this study, we found that oomycete *P. infestans* mycelium was damaged and had a rough and swollen appearance after being treated with Iturin A. All these changes in mycelial appearance were probably due to damage to the internal cell structure (Cui et al., 2016; Huiskonen, 2018). Next, our TEM results showed that the inhibited cell membrane was disrupted, the disordered organelles had irregular shapes, and a large area of cavitation even appeared in the cytoplasm center. All of the results above in our study were essentially the same as those in reports by Kruijt et al. (2009). In their experiments, lipopeptides putisolvin could also affect the oomycete pathogens *Pythium ultimum* and *P. capsici* through hyphal swelling and intracellular activity reduction. Additionally, previous reports indicated that the iturins family could disrupt the *B. cinerea* cytoplasmic membrane by creating transmembrane channels, which resulted in K^+^ leakage (Arrebola et al., 2010). In addition, the leakage of protein and nucleic acid caused by cell membrane damage could also change the relative conductivity of a mycelium-rich solution (Dolezalova and Lukes, 2015). Additionally, after treatment with the fluorescent dye propidium iodide, red fluorescence distinguished the damaged cell membrane from the intact membrane (Zhang and Sun, 2018). In our results, we demonstrated that Iturin A treatment could result in oomycete *P. infestans* cell membrane damage based on protein and nucleic acid release, increased conductivity of a mycelium-rich solution (two times), and obvious red fluorescence in sporangia.

Intracellular chaos caused by long-term inhibition stress can also induce ROS accumulation in cells (Huiskonen, 2018), and ROS accumulation is an important intermediate step in the progression of cell damage (Tian et al., 2018). For example, the CLP fengycin derived from *B. subtilis* BS155 had strong antagonistic activity against *M. grisea* involving ROS accumulation and MDA production (Zhang and Sun, 2018). In our study, after Iturin A treatment, the highest amount of ROS generation was four times that of the control, and the MDA concentration reached a maximum after 16 h of treatment. In harsh environments with ROS stress, *P. infestans* mitochondria might develop into an abnormal state and the cell respiratory process might be obstructed (Hinkle, 2004; Amaroli et al., 2019). Additionally, during oxidative stress, the accumulation of oxidized products could also cause MRCCA decline and electron transport chain dysfunction, resulting in an immature respiration process and a P/O decrease (Hinkle, 2004; Amaroli et al., 2019; Sun et al., 2019). In this research, we also demonstrated that Iturin A could lead to the mitochondrial dysfunction of oomycete *P. infestans* via MMP decrease and a more than 30% decline in the MRCCAs I-V. Additionally, after inhibition, the energy supply efficiency in *P. infestans* mitochondria determined by the RCR and P/O values was significantly lower than those of the control.

Through this study, we clarified that *B. subtilis* WL-2 can produce the CLP surfactin and Iturin A subfamilies, but surfactin had no direct inhibitory effect on *P. infestans* mycelium growth. Most importantly, we first found that Iturin A could suppress the oomycete *P. infestans* by cell structure disruption, oxidative stress, and energy supply dysfunction. All the results highlight that *B. subtilis* WL-2 and its Iturin A lipopeptide have great potential for inhibiting *P. infestans* mycelium growth and controlling the development of potato late blight. At present, many researchers have indicated that CLPs have an important role in controlling plant disease by causing the induction of systemic resistance (ISR) in plants against pathogens (Ongena et al., 2007); however, the induction of disease resistance and the differences caused by surfactin, iturin, and fengycin families in potato against late blight are still unknown and worth exploring.

## Author’s Note

This manuscript has been released as a pre-print at BioRxiv (Youyou Wang et al.).

## Data Availability Statement

All datasets contained in this study are listed in the manuscript.

## Author Contributions

YW and JJ contributed to the study conception and design. YW, CZ, and WG performed the material preparation, data collection, and analysis. YW, LW, and JL wrote the first draft of the manuscript. All authors commented on previous versions of the manuscript and read and approved the final manuscript.

## Conflict of Interest

The authors declare that the research was conducted in the absence of any commercial or financial relationships that could be construed as a potential conflict of interest.
