# Supplementary material for: Iturin A Extracted From Bacillus subtilis WL-2 Affects Phytophthora infestans via Cell Structure Disruption, Oxidative Stress, and Energy Supply Dysfunction
Source: Front Microbiol. 2020 Sep 9;11:536083. doi: 10.3389/fmicb.2020.536083 (PMC7509112; doi:10.3389/fmicb.2020.536083)
Supplement: Supplementary file 1 [file Data_Sheet_1.PDF]

Supplementary Material

Supplementary Figures

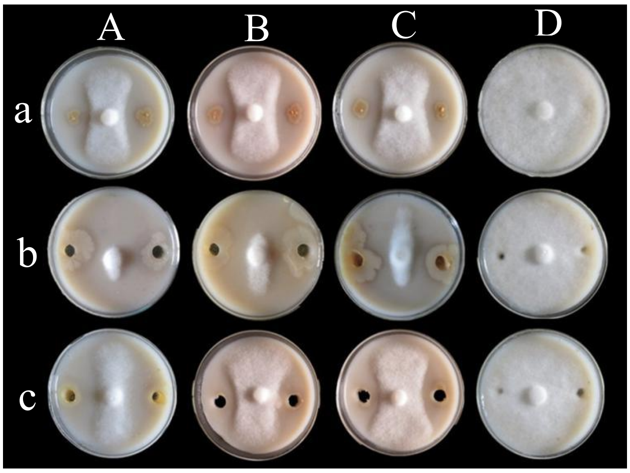

Figure S1. Comparison of the effects of the three strains on *P. infestans.* A: WL-2, B: WL-1, C: W-7, D: Control. a: Living cells (LCs), b: Cell suspension (CS), c: Cell-free supernatant (CFS). Photographs are representative of experiments performed in triplicate (the same below).

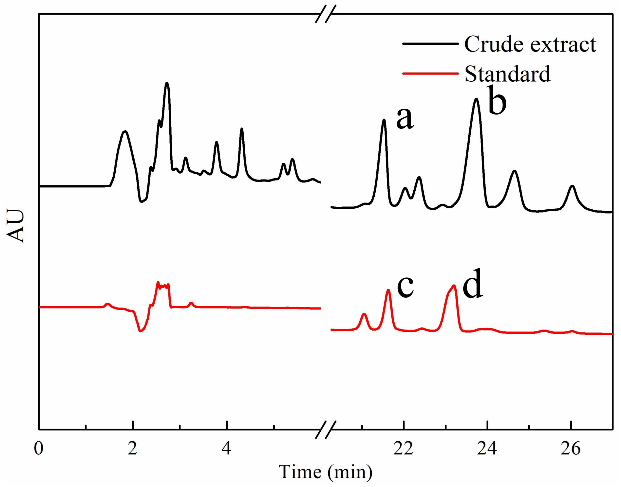

Figure S2. Purification of crude lipopeptide extract (CLE) from *B. subtilis* WL-2 using HPLC. The black line (top) shows the chromatogram for the CLE (peak a at 21.4 min and peak b at 23.6 min). The red line (bottom) represents the chromatogram for standard lipopeptides; peak c at 21.6 min is commercial surfactin, and peak d at 23.2 min is commercial iturins.

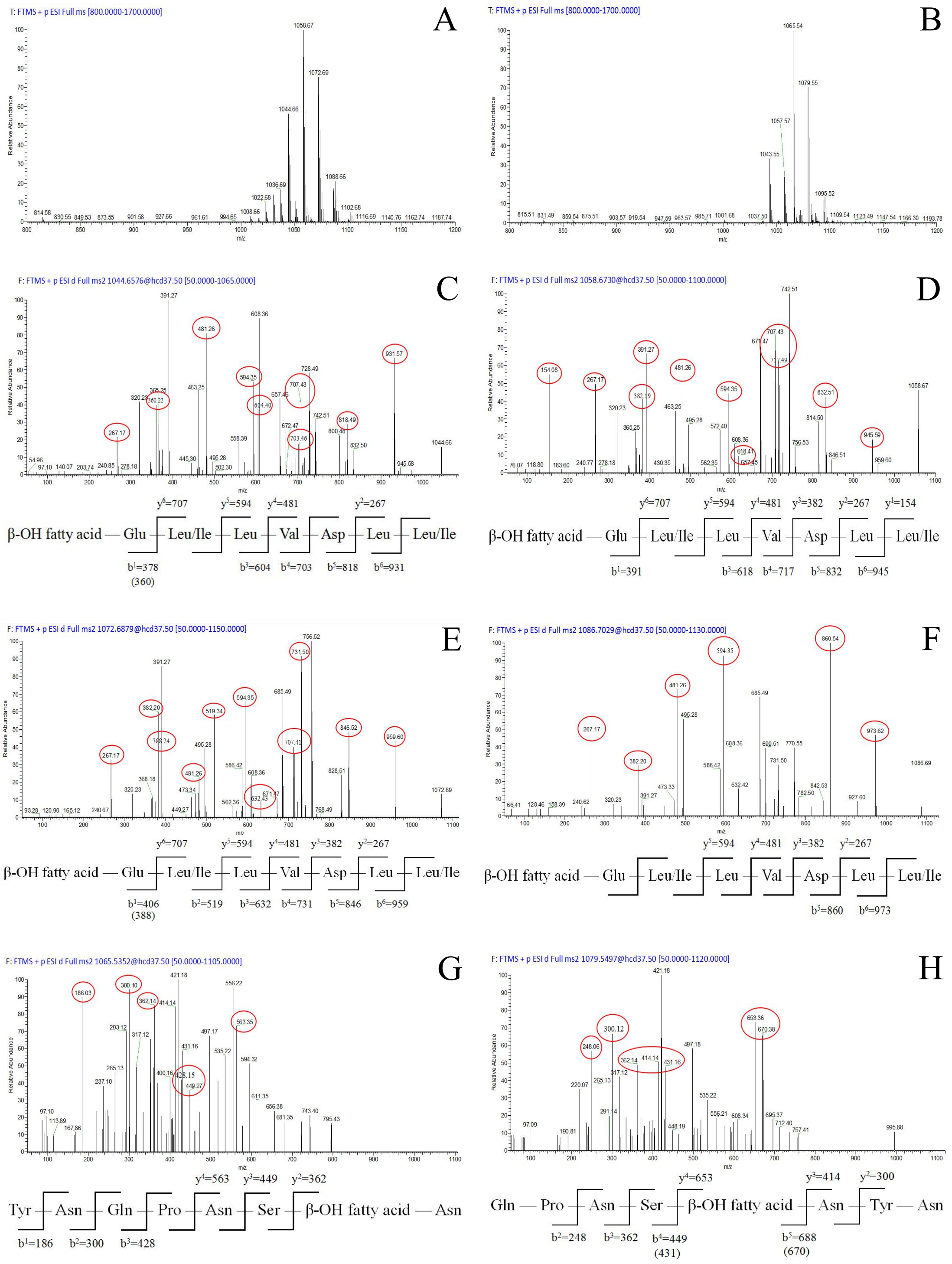

Figure S3. Detection of purified lipopeptides (surfactin and Iturin A) from *B. subtilis* WL-2 using MALDI-TOF-MS/MS. A: Full mass spectrum of peak a (surfactin); B: Full mass spectrum of peak b (Iturin A); C-H: MS/MS spectra of surfactin C_{14} (1,044.66), C_{15} (1,058.67), C_{16} (1,072.69), and C_{17} (1,086.69), Iturin A C_{14} (1,065.54), and Iturin A C_{15} (1,079.55) with Na^+ adduct ions.

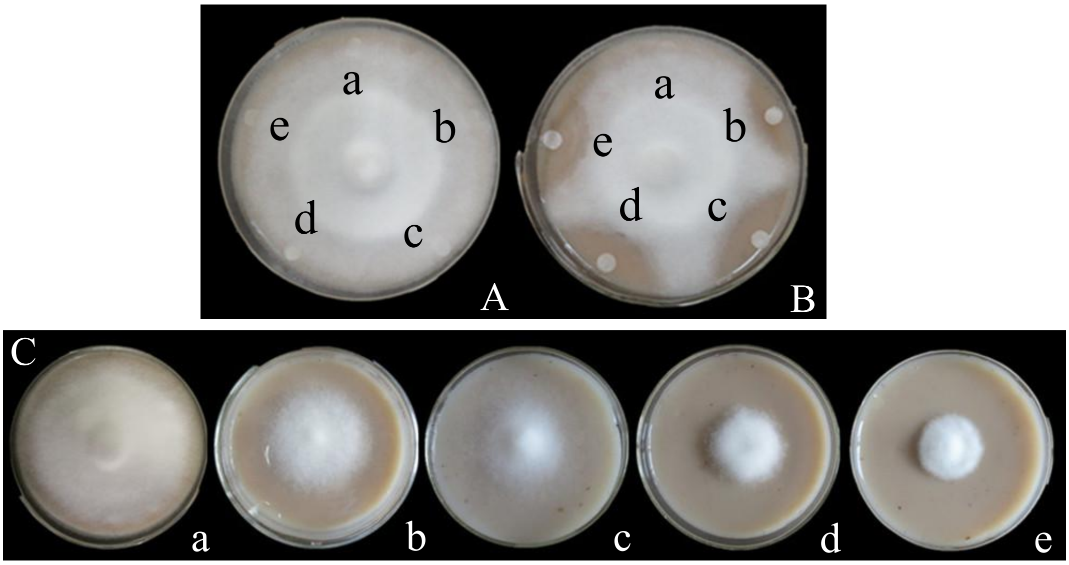

Figure S4. Inhibition effect of purified surfactin and Iturin A on *P. infestans* mycelium growth. A: Surfactin groups, B: Iturin A groups. C: Mycelium recovery growth after Iturin A inhibition. a: Control (distilled water), b-e: Drugs concentrations at 20, 30, 40, and 50 µg/mL, respectively.

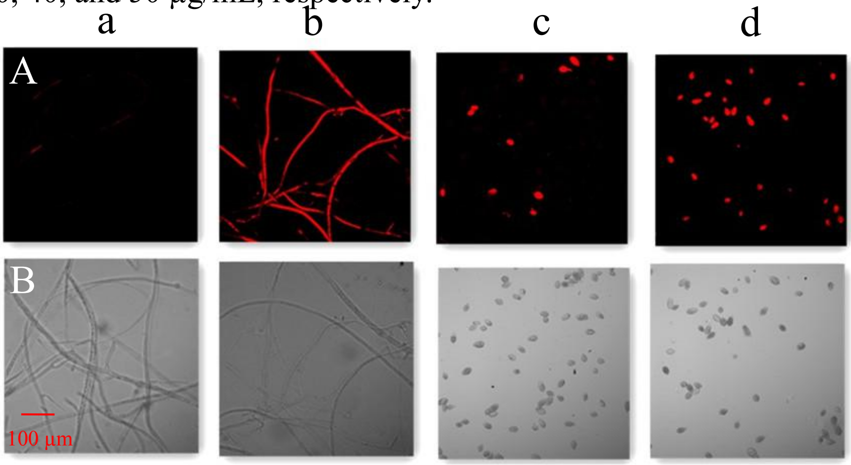

Figure S5. Effect of Iturin A (50 µg/mL) on *P. infestans* cell membrane integrity. A: Red fluorescence channel, B: Optical channel. a and c: Control groups, b and d: Treatment groups.

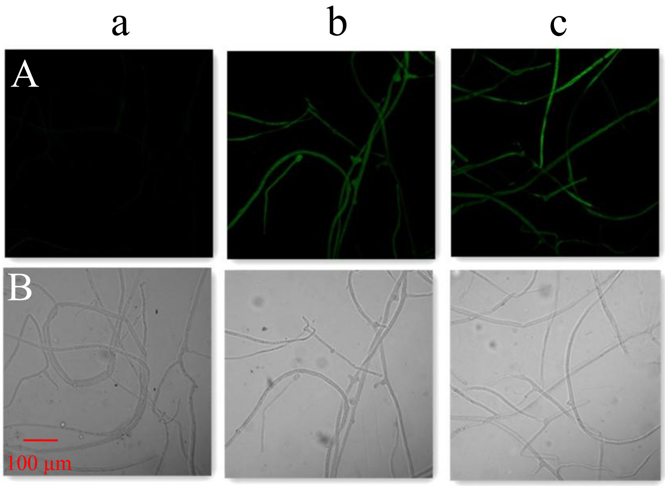

Figure S6. Reactive oxygen species (ROS) detection in *P. infestans* cell after Iturin A inhibition. A: Green fluorescence channel, B: Optical channel. a: Control without inhibition, b: Iturin A (50 μg/mL), generation for 16 h, c: Positive control (Rosup), 20 min after treatment.
